# Comment on the effectiveness of sodium hypochlorite against Clostridioides difficile spores

**DOI:** 10.1099/mic.0.001436

**Published:** 2024-05-21

**Authors:** Jennifer L. Cadnum, Claire E. Kaple, William A. Rutala, Curtis J. Donskey

**Affiliations:** 1 Research Service, Louis Stokes Cleveland VA Medical Center, Cleveland, Ohio; 2 Biomedical Sciences Training Program, Case Western Reserve University, Cleveland, Ohio; 3 Statewide Program for Infection Control and Epidemiology, University of North Carolina School (UNC) of Medicine, Chapel Hill, North Carolina; 4 Geriatric Research, Education, and Clinical Center, Louis Stokes Cleveland VA Medical Center, Cleveland, Ohio; 5 Department of Medicine, Case Western Reserve University School of Medicine, Cleveland, Ohio

**Keywords:** sodium hypochlorite, disinfectant, *Clostridioides difficile*, environment

**Dear Editor,**

Sodium hypochlorite and other chlorine-releasing disinfectants have been a mainstay of efforts to prevent transmission of *Clostridioides difficile* for decades [1]. Many chlorine-releasing products are registered by the United States Environmental Protection Agency (EPA) for use against *C. difficile* spores based on required laboratory testing data [2]. However, Ahmed and Joshi [3] recently reported that spores from three strains of *C. difficile* were minimally reduced after a 10 min exposure to sodium hypochlorite, although the preparation tested was not an EPA-registered sporicidal product and a standardized test protocol was not used [2]. This report and two other recent publications have raised concern that strains of *C. difficile* with reduced susceptibility to chlorine-releasing disinfects may be emerging [4,5]. To address this concern, there is an urgent need to test the effectiveness of EPA-registered chlorine-releasing agents against the isolates reported to have reduced susceptibility using a standard test protocol.

We used the American Society for Testing and Materials (ASTM) Standard Quantitative Carrier Disc Test Method (ASTM E-2197–02) to test the efficacy of an EPA-registered sporicidal disinfectant against *C. difficile* spores [6]. Five percent fetal calf serum was used as a soil load. The *C. difficile* isolates tested included the strain recommended by the EPA for testing of disinfectant efficacy against *C. difficile* (American Type Culture Collection [ATCC] 43598) [7], a clinical ribotype 027 strain from the Cleveland VA Medical centre (VA 17), and one of the strains tested by Ahmed and Joshi [2] (R20291, ribotype 027). Spores were prepared using the method recommended by the EPA for spore preparation [7]. The disinfectant tested was Clorox Pro Germicidal Bleach Concentrate (Clorox Healthcare, Oakland, CA) diluted with tap water to yield approximate concentrations of 500, 1000, 5000, 7850, and 10 000 parts per million (ppm) available chlorine; control carriers were inoculated with an equivalent volume of tap water. The EPA List K registration number of the product is 67619–32 with a listed contact time of 5 min; per the manufacturer, dilution of one part concentrate with nine parts water results in ~7800 ppm available chlorine with a 3 min contact time required to kill *C. difficile* spores. The approximate concentration of chlorine was confirmed using total chlorine test papers (Lamotte, Chestertown, MD). The pH of the diluted sodium hypochlorite solutions was ≥10. Testing was completed with exposure times of 1, 5, and 10 min in three separate experiments. The carriers were neutralized with Dey-Engley neutralizing medium (Remel Products, Lenexa, KS). Specimens were incubated on *C. difficile* brucella agar containing 5 mg l^−1^ of lysozyme [8]. Log_10_ reductions were calculated by subtracting viable organisms recovered after exposure to the disinfectant versus water controls.

Fig. 1 shows the log_10_ reductions for the three *C*. *difficile* test isolates. Sodium hypochlorite at 10 000 or 7850 ppm reduced spores of all three *C*. *difficile* isolates by ≥6 log_10_ with 5 and 10 min of contact time. At 5000 ppm, >6 log_10_ and >3 log_10_ reductions were achieved with the 10- and 5 min contact times, respectively. Minimal reductions occurred for the 1000 and 500 ppm concentrations for all three isolates.

**Fig. 1.**
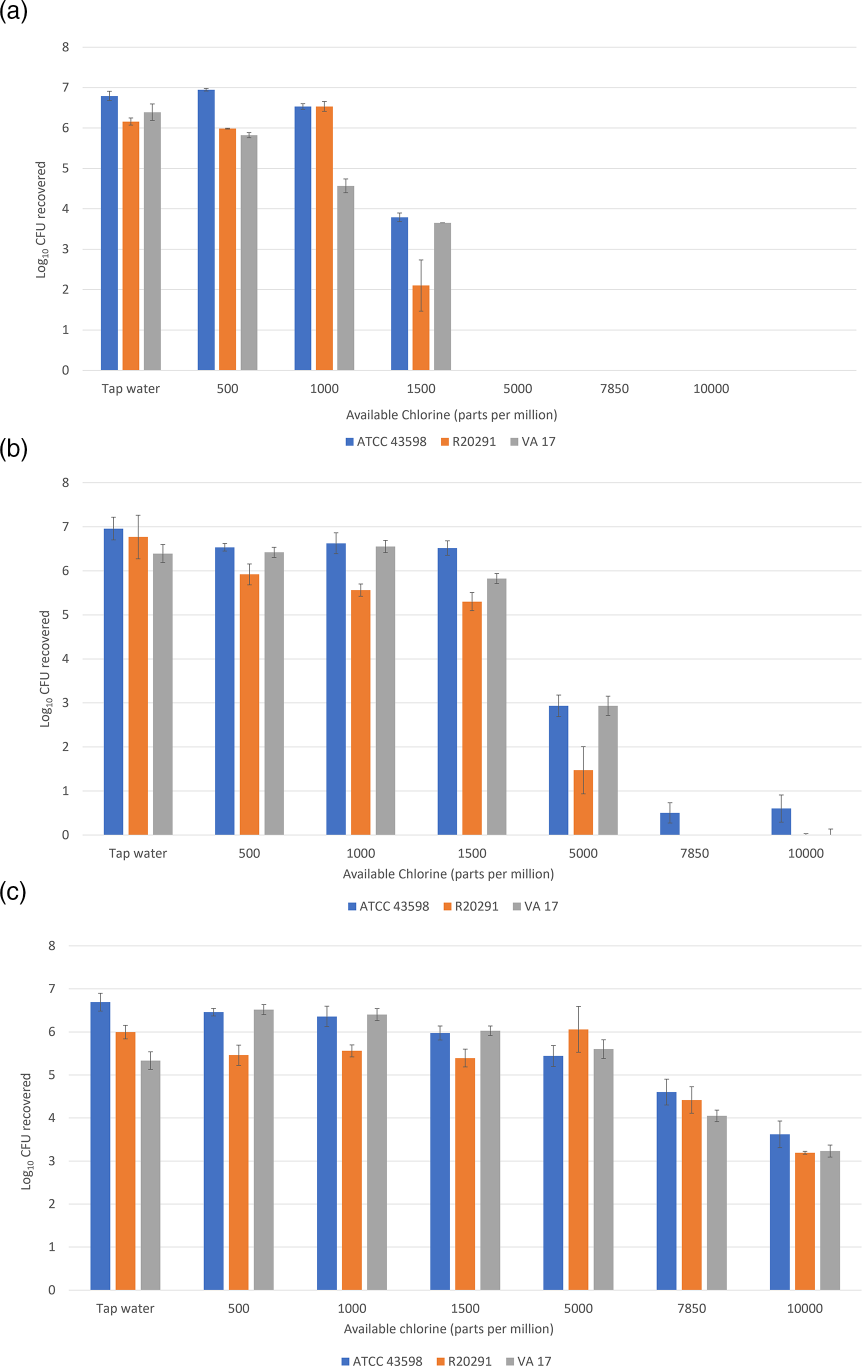
Mean (SE) log_10_ recovery of spores of *Clostridioides difficile* American Type Culture Collection (ATCC) 43598, VA 17 (a clinical ribotype 027 isolate), and R20291 (ribotype 027) after exposure times of 10 min (**a**), 5 min (**b**), and 1 min (**c**) using a standard quantitative disc carrier test method. Error bars represent standard error.

In summary, a commercial sodium hypochlorite product at 7850 and 10 000 ppm available chlorine reduced *C. difficile* spores by ≥6 log_10_ with five or 10 min of contact time but had limited efficacy at concentrations of 1000 and 500 ppm reduction or with a 1 min exposure time. The test strains included an isolate (R20291) recently reported to exhibit tolerance to disinfection with sodium hypochlorite [3].

The reason for the discrepancy between our findings and the recent reports suggesting reduced efficacy of chlorine-releasing agents is unclear [3,5]. The fact that the reported efficacy of chlorine-releasing agents against individual *C. difficile* isolates has varied considerably in different studies suggests that variations may be related to differing test agents (i.e. commercial product registered with the EPA as a sporicide versus a preparation not registered as a disinfectant) or test methods. For example, Table 1 shows variable results for spores of the R20291 isolate in several studies evaluating chlorine-releasing agents [3,5,9], with several studies demonstrating that chlorine-releasing disinfectants remain effective [9,11]. Given these considerations, more information on the sodium hypochlorite formulation (i.e. pH, measurements of free chlorine) and the test protocol used by Ahmed and Joshi would be helpful. Information on pH is of interest as reducing the pH may enhance the efficacy of chlorine-releasing disinfectants by increasing the proportion of hypochlorous acid versus hypochlorite ions [9,13]. Finally, given the high genomic fluidity of ribotype 027 *C*. *difficile* strains [14], we cannot exclude the possibility that the R20291 isolate tested by Ahmed and Joshi [3] has accumulated genetic changes resulting in reduced susceptibility to chlorine-releasing disinfectants. Genetic analysis is indicated if additional testing confirms that the isolate has reduced susceptibility to commercial, EPA-registered chlorine-releasing products.

**Table 1.** Studies evaluating efficacy of chlorine-releasing agents against *Clostridioides difficile* isolate R20291 (ribotype 027)

Reference (year)	Chlorine-releasing agent	concn	Method	Soil load	Contact time	Log reduction
9 (2011)	Na hypochlorite wipe	5000 ppm	Three-stage protocol for wipes	None	5 min	4.64 ±0*
10 (2011)	NaDCC	1500 and 5000 ppm	Suspension	None	30 min	5†
11 (2016)	Na hypochlorite (household bleach)	400, 1000, 5000, and 8000 mg l^−1^ free chlorine	Suspension in PBS (alkaline) or BHI-S (neutral pH)‡		15 min	PBS: >6 log reduction at all concentrations; BHI-S: <1 log reduction§
5 (2017)	NaDCC	500 ppm	Suspension	None	10 min	2.4||
12 (2019)	NaDCC	1000 ppm	Suspension and inoculated on steel, vinyl, and gown material	None	10 min	Suspension: >6 log reduction;On surfaces: >3 log reduction
4 (2023)	Na hypochlorite¶	5000 ppm	Suspension	None	10 min	<1
		10 000 ppm	Suspension		10 min	>3
3 (2023)	Na hypochlorite¶	1000–10 000 ppm	Suspension	None	10 min	<1

* nNo significant reduction with unmedicated control wipe alone (.42+0.07 log_10_ reduction).

† rReported as a 5 log_10_ reduction in the R20291 isolate with 0 % survival for 1500 and 5000 ppm available chlorine, but with surviving spores recovered at 500 and 1000 ppm available chlorine.

‡ BHI-S (pH 6.8) with pH of 6.04–6.6 after addition of bleach; PBS (pH 7.4) with pH 8.4–10 after addition of bleach.

§ tThe lack of reduction in BHI-S was attributed to the neutral pH.

|| tText states a 4 to 6 log reduction occurred for 21 different *C. difficile* isolates including R20291.

¶ Merck (105 614, Sigma Aldrich).

BHI-S Brain heart infusion-supplemented NaDCC sodium dichloroisocyanurate PBS phosphate-buffered saline

Our findings provide reassurance that EPA-registered sodium hypochlorite disinfectants remain effective against *C. difficile* spores, including an isolate recently reported to exhibit tolerance to sodium hypochlorite (R20291) [3]. In previous studies, EPA-registered chlorine-releasing products have been shown to be effective in reducing spores on surfaces in clinical settings [15,16]. Nevertheless, the work of Ahmed and Joshi is notable because it highlights the need for ongoing evaluations of chlorine-releasing disinfectants. Surveillance is needed to confirm that sporicidal disinfectants used in clinical practice remain effective against emerging strains of *C. difficile*. For efficacy against *C. difficile*, it is necessary to select products that provide sufficient available chlorine and apply them in a manner that provides adequate contact time. As noted by Ahmed and Joshi [3], there is concern that some commonly used disinfectants may provide suboptimal concentrations of available chlorine and require longer contact times than are likely to be achieved in real-world settings [3].

## Abbreviations

ASTM: American Society for Testing and Materials
ATCC: American Type Culture Collection
C. difficile: Clostridioides difficile
EPA: Environmental Protection Agency
NaDCC: Sodium dichloroisocyanurate
PBS: Phosphate buffered saline
SE: Standard error
